# Adverse reaction signal detection methodology in pharmacoepidemiology

**DOI:** 10.1007/s10654-018-0417-5

**Published:** 2018-06-04

**Authors:** Bruno H. Stricker

**Affiliations:** Department of Epidemiology, Erasmus Medical Center, P.O. Box 2040, 3000 CA Rotterdam, The Netherlands

In the nineteenth century, the toxicity of chloroform led to its withdrawal from clinical use [1] and in the period 1920–1940, hepatic injury by cinchophen [2] and agranulocytosis by amidopyrine and related agents [3] were recognized. But from the point of view of detection of important unknown adverse reactions, the thalidomide disaster with its thousands of fatal and non-fatal cases of congenital malformations was an absolute hallmark [4]. As a direct consequence, it was made mandatory in the early sixties of the preceding century to perform extensive toxicological, preclinical, and clinical studies before marketing of a drug in Western countries, and national spontaneous monitoring systems were set up. These systems in concert with the medical literature, proved to be the most effective and efficient system for recognizing new adverse reactions since then [5]. In the years thereafter, several drugs were recognized as the cause of serious disease, such as chronic active hepatitis by oxyphenisatin [6], sclerosing peritonitis by practolol [7] and many more since then. Such monitoring consists of manual review of adverse reaction reports by medical professionals and is relatively cheap and flexible but suffers from substantial underreporting, potential false-positive reporting and absence of reliable usage figures. Also, case-by-case assessments may lead to a loss of overview when large numbers of reports are involved and rests heavily on the quality of the professional.

In 1974, in an attempt to improve adverse reaction signal detection, Finney proposed to compare the proportion of reports of a certain drug-event association with the proportion of reports of that event to all other drugs in the database and test for significance in a 2 × 2 table [8]. A significantly higher proportion comprised a signal. A further extension of this principle with the magnitude expressed as a reporting odds ratio with 95% confidence limits was first proposed in 1992 [9], and as a proportional reporting ratio in 2001 [10]. Of these two effect measures, the reporting odds ratio has certain advantages [11]. These measures are now extensively used by the pharmaceutical industry as one of the tools of signal detection, in line with European guidelines [12]. But up till recently, the large majority of pharmaceutical marketing authorization holders only check their own database which is limited to those drugs which are marketed by that particular company. Only some of them also use the WHO Vigibase or the FDA Adverse Event Reporting System and since 2018, the Europeans Medicine Agency’s database Eudravigilance can be used.

A new development in signal detection is to use not only adverse reaction reports but complete medical records healthcare databases for this goal. Elsewhere in this journal, Hallas et al. [13] describe how a hypothesis-free screening of large administrative databases can be used for recognition of new drug-outcome associations. This is one of the examples of how the strong increase in computerization in the past decades and the consequent growth of automated healthcare data can be employed to this end. In current initiatives such as EU-ADR [14] and the Observational health data science and informatics (https://ohdsi.org/), networks of administrative databases were built to identify drug safety issues by data mining, mainly through a self-controlled design covering data from up to many millions of people. The question whether we should be happy with such a development is completely irrelevant. In human history, any technical development than can be used will be used. And data mining has proven very successful in genetic research. Genome-wide association studies (GWAs) by consortia of population-based cohort studies such as CHARGE were very rewarding in finding new associations between genetic variants and disease [15]. Especially in Western countries the combination of risk aversion and legislation is an enormous enforcer to employ such healthcare information for safety research and as long as the privacy of patients is guaranteed, there little against using it. But the consequences are that the number of false-positive signals that will have to be tested increases enormously. This requires a rigorous process of signal prioritisation and testing as the number of epidemiological resources is not endless. Apart from the subject itself, there are a number of important differences between data mining in genetic epidemiology and in pharmacoepidemiology. First, in genome-wide association studies Bonferroni corrections are used. There are many good arguments against using Bonferroni corrections whatsoever [16] but in GWAs they are the only workable solutions as using a *p* value of 0.05 as a cut-off would be very impractical in view of the abundance of associations when studying millions of single nucleotide polymorphisms. In data mining with healthcare databases the number of associations that can be tested is smaller and Bonferroni corrections are less commonly used, maybe also because of a fear of litigations for drug marketing authorization holders for missing associations. Second, GWAs in consortia often work with identical platforms. Healthcare databases are, however, very heterogeneous. Not only do they vary between countries and healthcare systems but also over time changes in insurance system and disease coding may complicate consistent analyses. Moreover, hospital-based and general practitioner’s healthcare information is structured in a different way, and mapping towards one analysable dataset is a cumbersome challenge which has to be repeated again and again. Third, and maybe most important, genetic GWAs are driven by scientific interest, rather than for fulfilling legal obligations. In how far this leads to better science remains to be seen. But one conclusion, we can make already now. If we do not improve our ability to distinguish true-positive from false-positive signals in an efficient way, we might waste epidemiologic resources for extensive signal-testing as a consequence of our increasingly demanding society.
